# Quality of Life, Depression, and Anxiety in Patients with Uveal Melanoma: A Review

**DOI:** 10.1155/2018/5253109

**Published:** 2018-03-20

**Authors:** Mario Miniati, Maria Grazia Fabrini, Federica Genovesi Ebert, Maricia Mancino, Alessandra Maglio, Gabriele Massimetti, Enrico Massimetti, Donatella Marazziti

**Affiliations:** ^1^Department of Clinical and Experimental Medicine, Section of Psychiatry, University of Pisa, 57 Via Roma, Pisa, Italy; ^2^Department of Radiotherapy, University of Pisa, 57 Via Roma, Pisa, Italy; ^3^Ophthalmic Surgery Department, University of Pisa, 57 Via Roma, Pisa, Italy; ^4^ASST Bergamo Ovest, Piazzale Ospedale 1, Treviglio, Italy

## Abstract

The aim is to summarize current knowledge on both QoL and depressive/anxious symptoms in patients with UM, including studies on the effect on QoL and psychological status of genetic testing related to the risk of metastatic disease. A review from the last 25 years by using the databases “PsycInfo,” “Medline,” and “Science Direct” was performed. As a total result, eighteen papers were retrieved. Eight studies (44.4%) used a prospective design methodology: two were retrospective observations (11.1%), three were cross-sectional observational studies (16.6%), and three (16.6%) were naturalistic follow-up studies. One trial was conducted with a case-control design (5.5%), and one was a methodological paper (5.5%). The number of subjects included in the studies ranged widely, between 7 and 842 (mean: 152.1 ± 201.3), for a total of 2587 patients, 1306 males (50.5%) and 1281 females (49.5%). The mean age of subject enrolled was 61.3 ± 4.1 years. Twenty-six different scales, questionnaires, or interviews were utilized. No significant differences in QoL between radiotherapy and enucleation emerged. Genetic testing did not significantly affect QoL or psychological status.

## 1. Introduction

Melanoma of the uveal tract is the most common primary intraocular malignancy in adults. The mean age-adjusted incidence of uveal melanoma (UM) in USA is approximately 4.3 new cases for 1.000.000 people per year [1]. Mortality varies by cancer size and age, ranging between 35% and 50%, five and ten years after enucleation. Men have a higher incidence than women (4.9 versus 3.7 per million) [1]. The treatments for UM are surgery and radiotherapy, and several studies of the past had a primary aim to determine whether eye sparing brachytherapy offered patients the same chance of survival of enucleation [2]. The issue of whether enucleation or radiotherapy should be used has been originally addressed by two multicenter randomized clinical trials included in the Collaborative Ocular Melanoma Study (COMS), in which survival rates between patients treated with enucleation and those treated with radiation for medium-sized melanoma were compared [3]. The primary outcome was the overall survival. The secondary outcome included cancer-free survival and visual acuity. The original design of the COMS did not consider the evaluation of psychological or psychopathological consequences of diagnosis and treatments, nor the evaluation of quality of life (QoL). COMS generated a number or reports, since 1990 [3]. An ancillary component of the COMS, the COMS Quality of Life Study (COMS-QOLS), was subsequently designed to measure the impact of choroidal melanoma on QoL, with the first report published in 1999 [4]. The assessment included the emotional and physical outcomes and the social role functions. However, other psychological aspects remained largely unexplored, such as depressive or anxiety disorders. Usually, in UM studies, anxiety and depression have been considered as originating from the presence of the oncologic disease and, for this reason, interpreted as direct consequences of physical suffering [5].

The aim of this paper is to summarize current knowledge on both QoL and depressive/anxious symptoms in patients with UM, including studies on the effect of genetic testing related to the risk of metastatic disease on QoL in patients who have already been diagnosed with UM. Methodological issues regarding experimental study designs, diagnostic procedures, and outcome measures are raised.

## 2. Materials and Methods

A literature review from the last 25 years was conducted by using the databases “PsycInfo,” “Medline,” and “Science Direct,” with the following keywords: “Choroidal” AND “melanoma” AND “quality of life” [MeSH Terms] OR “uveal” and “melanoma” AND “quality of life” [MeSH Terms], OR “COMS” AND “QOLS” AND “research report” [MeSH Terms] OR “research” AND “report” OR “research report” [MeSH Terms] OR (“genetic” [All Fields] AND “testing” [All Fields]) OR (“genetic testing” [All Fields]) AND (“Uveal melanoma” [Supplementary Concept] OR “Uveal melanoma” [All Fields] OR “uveal melanoma” [All Fields]). Papers not in English were excluded as well as literature reviews or study protocol papers. As a total result, 18 papers were selected.

## 3. Results

The main characteristics of the 18 studies are listed in Tables 1(a) and 1(b). The heterogeneity of sample compositions, treatment, settings of treatment, and analyzed predictors did not permit carrying out a meta-analysis of all of the available studies. Eight studies (44.4%) used a prospective design methodology: two were retrospective observations (11.1%), three were cross-sectional observational studies (16.6%), and three (16.6%) naturalistic follow-up studies. One trial was conducted with a case-control design (5.5%), and one was a methodological paper (5.5%). The number of subjects included in the studies ranged widely, between 7 and 842 (mean: 152.1 ± 201.3), for a total of 2587 patients, 1306 males (50.5%), and 1281 females (49.5%). The size of the samples and the diagnosis composition depended on the characteristics of the setting and on the duration of observation. The mean age, according to the papers that reported this variable, was 61.3 ± 4.1 years. With regard to psychiatric diagnoses, no studies utilized standardized diagnostic criteria such as the ones described in the Diagnostic and Statistical Manual of Mental Disorders (DSM-IV-R or previously DSM-III-R) [6, 7]. Details on rating scales administered are summarized in Table 1(b). The Hospital Anxiety and Depression Scale (HADS) was administered in seven studies (38.8%) and the Beck Depression Inventory (BDI) in only one study (5.5%) [8, 9]. The European Organization for Research and Treatment of Cancer Questionnaire (EORTC-QLQ) was administered in 4 studies, but in different versions (22.2%) [10, 11]. The MOS-SF-36 was utilized in 8 studies (44.5%) [12].

### 3.1. Quality of Life (QoL) and Depression/Anxiety Symptoms Assessment

Quality of life (QoL) has been originally assessed in a multicenter ancillary study of the COMS and the COMS-QOLS [3, 4], designed to measure the impact of CM diagnosis and treatment on QoL, comparing enucleation to radiation therapy. COMS consisted of two multicenter clinical trials for large and medium CM and a third arm on the natural history of small CM. Patients with large CM (>8 mm in thickness and/or >16 mm in longest base diameter) were randomized to enucleation alone or enucleation after an external-beam radiation (20 Gy). Patients with medium CM (3.1 to 8 mm in thickness and no more than 16 mm in longest base diameter) were randomized to enucleation or brachytherapy using iodine-125. Patients with small CM (1 to 3 mm in apical thickness and at least 5 mm in diameter) were enrolled in a registry and followed up. The primary outcome was time to death from all-cause mortality. Secondary outcomes included metastasis-free survival, cancer-free survival, and years of useful vision. Patients were randomized to either enucleation or enucleation preceded by external-beam radiation. Patients with medium CM were offered to participate the QOLS. A total of 209 (79%) out of 265 patients participated in the QOLS assessments. Considering that there were no differences in 5-year all-cause mortality for large- and medium-size choroidal melanomas, the subjective perception of QoL became a key issue for the treatment selection. The COMS-QOLS assessment included the Medical Outcomes Study (MOS) 36-item Short-Form Health Survey (MOS-SF-36), the Activities of Daily Vision Scale (ADVS), the National Eye Visual Functioning Questionnaire (NEI-VFQ), and the Hospital Anxiety and Depression Scale (HADS) [8, 12–14]. The internal consistency of the MOS-SF-36 was validated for patients with CM in this study [15]. Patients were evaluated at baseline, after six months, and annually. Several reports were published [3, 16, 17]. The 2006 report focused on the comparison between the group randomized to enucleation (*n* = 106) and the group randomized to brachytherapy (*n* = 103) [17]. The HADS anxiety scores were analyzed over time by diagnostic category (“no anxiety” versus “possible or definite anxiety”). Patients randomized to brachytherapy with symptoms of anxiety were less likely to report resolution of symptoms than patients randomized to enucleation, but the proportion of patients with “definite anxiety” did not differ by treatment group. The levels of anxiety decreased after six months as compared with baseline. Similarly, depression scores on HADS did not differ by treatment. The choice of treatment was not associated with significant differences in both QoL and depression/anxiety levels in the long-term follow-up. This finding was confirmed in other studies. Cruickshanks and Colleagues [18] evaluated a sample of one hundred and forty-seven patients treated with enucleation or radiation therapy, using the Medical Outcome Study-MOS-SF-36, the National Eye Visual Function Questionnaire-NEI-VFQ, and the Time-Trade-off Interview [19]. Only two differences emerged in the QoL measures between patients treated with surgery and those treated with radiation therapy, on the “vitality” domain and on the “standardized mental component” of the MOS-SF-36. However, the scores of these domains were no longer statistically different at the end of follow-up.

In a 1-year follow-up study on a sample of 98 patients enrolled upon UM diagnosis confirmation, patients were categorized as suffering for clinical levels of anxiety or depression according to HADS scores [20]. QoL and the presence of a “psychological reaction” at the time of the diagnosis, 2 and 12 months after treatment with enucleation or ruthenium plaque radiotherapy, were assessed with the European for Research and Treatment of Cancer Questionnaire (QLQ-C30), the Impact of Event Scale (IES), and the Eye Symptom Questionnaire (validated within the study) [21]. No statistically significant differences between the treatment groups were found. More than 43% of the sample scored in the “borderline to pathologic” ranges for anxiety, and 19% for depression. Symptoms of anxiety, but not of depression, decreased at two months and one year after treatment. No specific psychiatric treatment or psychological support was considered even for those patients with clinically significant levels of distress. These studies utilized instruments not specifically constructed and validated for patients with CM, such as the MOS-SF-36 that was unable to detect signs and symptoms related to a single eye disease, or the VF-14 cataract symptom score, and the Vision Related Sickness impact profiles that were exclusively focused on visual function [22–25]. Foss and Colleagues (2000) addressed this issue with the validation of a short patient based questionnaire, the “21-item Measure of Outcome in Ocular Disease” (MOOD) [22], in a sample of 176 patients with inhomogeneous age range (from 22 to 86 years) and enrolled even if treated within a very extended time interval (1 to 197 months before), thus limiting the generalization of results. The MOOD is no longer used.

A case-control study compared 93 patients with UM who underwent radiotherapy versus 575 controls matched for age and gender [26], with the MOS-SF-36, the Symptom Checklist 90 Revised (SCL-90-R), and the Short Form of the Questionnaire for Social Support (K22) [27, 28]. As expected, patients were reported to be significantly poorer in global QoL (MOS-SF-36) than controls. Conversely, patients and controls differed neither as to the “Global Index of Social Support” (K-22) nor as to the 3 global indices and 9 subscales of the SCL-90-R. Thirty-three patients were diagnosed as “mentally distressed” and compared with those who were “mentally nondistressed” (*n* = 60) and with controls. Distressed patients were reported to be higher on “bodily pain” and significantly poorer in “mental health” both on the sum score level and on the subscale level, compared with nondistressed patients. Moreover, mentally distressed patients had a poorer QoL on all subscales (except for “social functioning”) and on global scores than controls. In a second study by the same authors, QoL was assessed in a sample of 35 patients (19 males and 16 females) affected by CM and treated with ruthenium radiotherapy [29]. Patients were evaluated immediately before and 3 months after treatment, compared to healthy controls (HC) and to patients with different oncologic/ophthalmologic diagnoses. Patients were considered at risk of “clinically relevant distress” according to the SCL-90-R definition (Global Severity Index and/or two subscales) if the T-score was ≥63. No information was provided on the course of anxious or depressive symptoms, despite the endorsement of the SCL-90-R “clinically relevant distress” threshold, in 49% of the sample before radiotherapy and in 31% three months after radiotherapy.

A previous retrospective study included 130 patients with UM treated with 3 different methods of radiotherapy, between 1988 and 2001 [30]. The 50% of the sample (*n* = 49) was treated with the stereotactic external-beam irradiation (SEBI) with a 6 MV linear accelerator (LINAC), while the 19.4%  (*n* = 19) was treated with the Ruthenium-106 brachytherapy, and the 21.4%  (*n* = 21) with the Leskell Gamma Knife therapy (GK). Seven patients (7.1%) underwent a secondary enucleation because of treatment failure or complications. QoL was assessed with questionnaires mailed to patients. Ninety-eight patients with UM treated with radiotherapy approximately 3 years before entering the study returned the questionnaires. Three areas were investigated: (a) QoL assessed with a visual analogue scale (VAS) administered before and after treatment; (b) physical symptoms and treatment side effects, measured with the EORTC QLQ-C30 and the EORTC QLQ-OPT30 questionnaires; (c) anxiety and depression signs and symptoms as a result of the psychological effects of illness and of the side-effects of therapy, measured with the Hospital Anxiety and Depression Scale (HADS) [31]. The level of QoL as individually experienced reached a mean of 74.1% before therapy and a mean of 69% after therapy, indicating a limited decline (5%), as confirmed by the EORTC-QLQ-OPT37 scores. The 23.7% of the sample scored in the “borderline to pathologic range” on HADS anxiety and depressive symptoms compared to 11.4% in nonclinical samples. Severe anxiety scores were recorded for 12.4% of patients. Severe depression scores were rated for 14.4% of patients. When comparing patients treated with the different forms of radiotherapy, no therapy-specific differences were found on HADS scores or on EORTC-QLQ-C30 and EORTC-QLQ-OPT37. When compared with those who had undergone secondary enucleation, results showed that the latter reported more fears of recurrences. The impact of enucleation on the posttherapeutic QoL was not significantly different between the two groups. No specific support to patients with significant levels of anxiety or depression was proposed (12–14% of the sample), and no questions to the need for psychiatric or psychological treatments were raised.

Blanco-Rivera and Colleagues (2008) [32] evaluated 65 patients (29 males and 36 females) with a questionnaire on visual function; the 14-item Visual Functioning Index (VF-14) modified to a 19 items version (VF-19) to determine whether brachytherapy or enucleation was better for QoL in patients who had a monocular involvement treated during the last six months with brachytherapy I 125, enucleation, or both. Forty-five patients were treated with brachytherapy, 14 patients were enucleated, and 6 patients were treated first with radiotherapy and later enucleated. There were no statistically significant differences between patients treated with radiotherapy and those treated with enucleation in terms of decrease in vision. Conversely, in terms of subjective QoL, the decrease in the VF-19 scores before and after treatment (3.89 ± 0.24; 3.66 ± 0.30, resp.) was statistically significant (*p* < 0.001). The difference between the enucleated and radiated groups was also statistically significant in favour of the last one (*p* = 0.008). There were statistically significant differences (*p* = 0.002) between the general states of health that radiated patients had as opposed to the enucleated. Major limitations of this study were that no additional rating scales were administered, and no assessment of depressive symptomatology was carried out, even if patients were followed-up for a long period (5 years). Moreover, 15 patients out of 65 (23% of the sample) were interviewed by telephone instead of being directly evaluated.

Depression has been investigated in a recent prospective study on a sample of patients with UM after surgical treatment (*n* = 20) [33]. The aim of the study was to perform a psychological assessment of patients with UM who have been referred for enucleation. Patients were evaluated upon diagnosis and referral for surgery, within 3 months after surgery and within 12 months after surgery, with the MOS-SF-36 and the Beck Depression Inventory (BDI). The overall sample of 20 patients showed a depressive state from “minimum” (55%; *n* = 11) to “mild” (35%; *n* = 7) to “moderate” (10%;  *n* = 2). After 3 months, all patients were still depressed (from “minimum state of depression” to “severe” in two patients). After 1 year, 14 of the 16 completers (87.5%) scored the “minimum” state of depression and two the “mild” state. The worse period from a psychological perspective occurred 3 months after surgery and at 1-year follow-up depressive symptoms, QoL, and social functioning improved.

In a recent longitudinal study, both QoL and signs and symptoms of anxiety and depression were assessed in a sample of 69 patients with CM treated with radiotherapy [34]. The study encompassed 4 evaluations: before treatment and 1 month, 6 months, and 12 months after treatment. Published data were on the assessment before treatment and after 1 month. Patients were evaluated with the EORTC-QLQ-C30, the EORTC-QLQ-OPT-30, the HADS, and the STAI-B (State-Trait Anxiety Inventory) [35]. Patients who completed the 1-month assessment were fifty-two. More than half of the patients (56%) showed moderated levels of anxiety, according to both HADS and STAI-B before the beginning of the treatment, with a statistically significant decrease at 1 month (from 8.45 ± 4.23 to 7.33 ± 4.43; *p* < 0.009). The depressive symptoms remained stable one month after treatment. The QoL remained relatively good and stable with the exception for social functioning that decreased after treatment.

A retrospective study on a sample of 99 patients who were treated for a CM 5 years before, either with enucleation or radiotherapy (proton beam therapy or brachytherapy), explored QoL, depressive symptoms, and concerns about recurrence [36], with the Cancer Needs Questionnaire-Short Form (CNQ- SF), the 25-item National Eye Institute Visual Function Questionnaire (NEI-VFQ), and the Center for Epidemiologic Studies Depression Scale (CES-D) and by the Concern about Recurrence Scale that explored how often patients thought about their CM coming back or spreading (metastasizing) and how upsetting they found these thoughts [37]. In a full regression model for QoL outcomes, patients who received enucleation had 0.57 standard deviation (SD) lower scores on the NEI-VFQ “role limitations” thus indicating more morbidity. Only the 15% of the sample fulfilled the CES-D cut-off (total score ≥ 16) for a clinically relevant depression, which was lower than reported in other studies on oncology samples, maybe because the number of patients treated with surgery was limited (16/99). The concerns about recurrences were high, even if no differences were found between treatments. This finding should be interpreted with caution due to the small number of patients receiving enucleation. The major limitation of this study was its retrospective nature, which excluded patients who died in the meantime.

QoL and the occurrence of depression and anxiety symptoms have been assessed also in relationship to the availability of UM genetic prognostic testing. As summarized in a recent review on this topic [38], there are two biological classes of UM, which significantly differ from each other on the metastasis risk. The genetic prognostic testing can be determined either through detection of monosomy 3 in UM DNA [39] or by a multigene expression profile of RNA [40]. Due to the UM class, the risk of developing metastases varies pronouncedly: the mortality rates due to metastasis were 13.2% for UM with disomy 3 and 75.1% for UM with monosomy 3 (median follow-up time of 5.2 years) [38]. Two studies, both published on 2009, were concordant on the finding that psychological status did not vary negatively as a function of cytogenetic test results [41, 42]. Studies were similar and mainly focused on the possible regrets for patients who had prognostic information with no substantial possibilities of changing the outcome. The most common reaction to the information provided was that the result of the cytogenetic testing would enable the patient* “to bring his/her life in control,”* even with a poor prognosis, as confirmed by a following study by Cook et al. [43] on patient autonomy with the practice of informed consent to the genetic testing. No high regret scores or clinically relevant depressive/anxious symptoms were also detected in a more recent prospective study by Schuermeyer et al., on a sample of patients with UM who accepted to participate in a program of prognostic testing. Patients with a high risk genotype were offered inclusion in a systemic adjuvant trial and were evaluated with the HADS and a modified version of the Decision Regret Scale (DRS) [44] immediately prior to treatment and 3 months and 12 months following the UM treatment [45]. For the first time, this study examined the possible coexistence of and the potential confounding role of depression and anxiety symptoms with decision regret. Results were partially in line with the previous studies. Patients were showing more anxiety and depression symptoms at baseline, with a reduction over time. The number of patients with depressive symptoms was very low at baseline (9%) but those who regretted their decision on cytogenetic testing were around 10%, with a DRS score significantly associated with the baseline HADS depressive score, suggesting a possible confounding role of depressive (not of anxious) symptoms on regrets.

A prospective longitudinal study by Hope-Stone et al. [46] compared the mean Functional Assessment of Cancer Therapy-General (FACT-G) scores [47] and the HADS scores of 411 patients with UM with the normative values of the same scales in an already published population, at 6 months, 1 year, and 2 years after treatment, testing the association of these scores with gender, age, enucleation, and monosomy 3. The study confirmed the absence of significant differences in outcomes at any point depending on whether patients were enucleated or not. Patients diagnosed with monosomy 3 were* “more depressed”* than others at each time point, with no decrease over-time (as reported in the previous studies), but again the HADS mean score was always below the cut-off scores for* “clinically relevant”* depression.

## 4. Discussion

QoL in patients with UM has been for the first time systematically evaluated in the COMS-QOLS studies. After the COMS-QOLS experience, the assessment of QoL became an important criterion in making medical decisions and the first step to involve patients in the process of treatment selection. Unfortunately, despite the growing interest in this specific field, results of the present review confirm the paucity of empirical evidence on the psychiatric evaluations of patients with UM. Only a limited number of reports had to deal with the subjective experience of patients with UM from a psychiatric point of view, even if the perception of QoL had become a key issue for treatment selection, instead of outcomes such as the time to death from all-cause mortality or the metastasis-free survival, the cancer-free survival, and the years of useful vision.

Research suggests that patients with UM have worse QoL than age-matched populations. Differences in QoL by treatment modality are small or absent. With the exception of treatment modality, little research has examined potential risk and protective factors for QoL in UM. Therefore, challenges to the identification of evidence-based finding are discouraging. Available studies are affected by several methodological limitations. The overall strength of the available evidence is poor, with a lack of consensus and inconclusive outcomes. As a consequence, impressions about “positive” and “negative” findings were shaped by single studies with a limited number of participants and often extrapolated across patient groups of different age, illness duration, and severity. One of the main limitations for the generalization of study results is that QoL in the special population of patients with UM has been explored with instruments, such as the EORTC QLQ-C30 that investigates general factors related to subjective well-being. Studies using disease-focused instruments (e.g.,, the VF-14) built and validated to measure visual problems caused by specific conditions (such as cataract) are unable to capture the complexity of the subjective changes. Taken as a whole, the number of instruments utilized for the assessment of QoL is too wide and inhomogeneous. In this review, 19 different scales were found in a total of 16 studies, raising questions on how to compare findings from such a number of scales, exploring in different ways different areas. The hypothesis that the diagnosis and treatment of UM could be associated with the presence of depressive and anxious symptoms is largely unexplored. Few specifically designed studies are available, and information derives mainly from studies on QoL, where depressive or anxiety symptoms were assessed aside. The only instrument utilized to assess the presence/absence of anxiety and depressive symptoms is the HADS, a scale not widely used for diagnostic purposes by psychiatrists, who prefer to utilize separate scales for two psychopathological areas who might be present at the same time but that are significantly different in terms of clinical presentation, outcomes, and duration. No other scales were administered to those patients, who reached the threshold for an anxiety or a depressive disorder with the HADS, and no diagnosis was actually made or pharmacotherapy/psychotherapy adopted. The most recent studies on genetic testing of UM confirm this trend. Although the therapeutic options available for metastatic UM are still limited, studies are concordant in finding that patients usually request the prognostic information about their UM at the time of diagnosis, in order to better plan their management, with no significant impact on their depression/anxiety levels nor on their subjectively perceived QoL, even when monosomy 3 is confirmed. Only the most recent studies on genetic testing offer patients an assessment with a psychologist during the perioperative period. Still no psychiatric assessment or psychopharmacological treatments are usually considered for patients with conditions that might reach clinical significance from a psychiatric point of view, raising questions on the rationale of not considering a more integrated approach (psychological and psychopharmacological) for a population of patients who are facing several challenge to their well-being associated with the disease, with its treatment, and with its prognostic evaluation.

## 5. Conclusions

Taken as a whole, QoL assessment did not reveal relevant differences among UM treatment groups in terms of overall QoL or depression/anxiety levels. Genetic testing did not significantly affect QoL or patients' psychological status. At present, there are limitations and gaps, from a psychopathological perspective, on several main topics, such as the occurrence of specific patterns of depressive/anxiety symptoms, as well as their treatment responses in the population of patients with UM. A more accurate definition of QoL that takes into account a psychiatric evaluation of anxiety and depression signs and symptoms is a key issue. Further research is necessary to define the most appropriate psychological/psychopathological assessment of QoL and depressive/anxiety symptoms in patients with UM.

## Figures and Tables

**(a):** 

Authors	Study design	Evaluation time	Pts #	Treatment 1	Treatment 2	Treatment 3
Moy & Melia, 1999 [4]	Methodological paper	6 months and annually after randomization	-	Brachytherapy	Surgery	
Cruickshanks et al., 1999 [18]	Cross-sectional	One evaluation after 4.9–6.3 ys	147	Brachytherapy	Surgery	
Brandberg et al., 2000 [20]	Naturalistic FU	12 months	99	Brachytherapy	Surgery	
Foss et al., 2000 [22]	Validation study	-	176	Brachytherapy	Surgery	Proton beam radiotherapy
Melia at al., 2003 [16]	Cross-sectional	-	842	Brachytherapy	Surgery	
Chabert et al., 2004 [30]	Retrospective	4	98	Brachytherapy	SEBI external beam	Leksell
Reimer et al., 2003 [26]	Naturalistic FU	3	35	Brachytherapy	-	
Melia et al. 2006 [17]	Naturalistic FU	60	209	Brachytherapy	Surgery	
Reimer et al., 2006 [29]	Case control	1 month	93	Thickness assigned	-	
Blanco-Rivera et al., 2008 [32]	Prospective	120	65	Brachytherapy	Surgery	
Cook et al., 2009 [41]	Prospective	4	14	Surgery	-	-
Beran et al., 2009 [42]	Longitudinal	-	99	Brachytherapy	Surgery	Proton beam radiotherapy
Amaro et al., 2010 [33]	Prospective	12	20	Surgery	-	
Suchocka-Capuano et al., 2011 [34]	Prospective	12	69	Brachytherapy	SEBI external beam	
Cook et al., 2011 [43]	Prospective	36	22	Brachytherapy	Surgery	
Wiley et al., 2013 [36]	Retrospective	60	99	Brachytherapy	SEBI external beam	Surgery
Schuermeyer et al., 2016 [45]	Prospective	12	96	Brachytherapy	Surgery	
Hope-Stone et al., 2016 [46]	Prospective	24	411	Brachytherapy	SEBI external beam	Surgery

**(b):** 

Authors	Outcome measure 1	Outcome measure 2	Outcome measure 3	Outcome measure 4	Outcome measure 5	Comments
Moy & Melia, 1999 [4]						Methodological paper.

Cruickshanks et al., 1999 [18]	MOS-SF-36	NEI-VFQ	TTO			Patients treated with radiotherapy had better scores on the Mental Component Subscales of MOS-SF-36. No other differences were found

Brandberg et al., 2000 [20]	HADS	EORTC-QLQ-C30	IES	ESQ		Symptoms of anxiety, but not of depression decreased at 2 months and 1 year after treatment. “Emotional problems” more represented among enucleated pts

Foss et al., 2000 [22]	MOS-SF-36	MOOD				The aim was to develop a measure to assess outcomes in patients treated for ocular melanoma (MOOD). The MOOD proved to be highly acceptable.

Melia et al., 2003 [16]	ADVS	MOS-SF-36	NEI-VFQ	HADS		

Chabert et al., 2004 [30]	HADS	EORTC-QLQ-C30	EORTC-QLQ-OPT-C37	VASs		QoL and state of health experienced by patients both seemed to be rather good. QoL after treatment did not appear to depend on the type of treatment. 7 pts were secondary on surgery after radiotherapy.

Reimer et al., 2003 [26]	SCL-90-R	MOS-SF-36	NEI-VFQ-42			No information regarding anxious or depressive symptoms, despite the endorsement of the SCL-90 clinically relevant distress threshold, in 49% of the sample before radiotherapy, and in 31% three months after radiotherapy.

Melia, 2006 [17]	ADVS	NEI-VFQ-42	MOS-SF-36	HADS	NSD-COMS-QoL	HADS anxiety scores were analyzed over time by categories (“no anxiety” vs “possible or definite anxiety”). Patients randomized to brachytherapy with symptoms of anxiety were less likely to report resolution of symptoms than patients randomized to enucleation. The proportion of patients with “definite anxiety” did not differ by treatment group. The levels of anxiety decreased after 6 months as compared with baseline. Depression scores on HADS did not differ by treatment

Reimer et al., 2006 [29]	SCL-90-R	MOS-SF-36	SFQSS-K22			Thirty-tree pts were “mentally distressed”, 60 pts were “non-distressed”. Subscales with scores > 63 in distressed patients included “Somatization”, “Anxiety”, and “Phobic anxiety” as well as the global scores Global Severity Index and Positive Symptom Distress Index.

Blanco-Rivera et al., 2008 [32]	VF-19					Statistically significant decrease in the VF-19 scores before and after treatment (3.89 ± 0.24; 3.66 ± 0.30, respectively; *p* < 0.001). The difference between the enucleated and radiated groups was statistically significant in favor of the last one (*p* = 0.008).

Cook et al., 2009 [41]	Detailed interview audio-recorded					According to authors' clinical impression, patients with good prognosis were the ones who benefit most from cytogenetic testing. No standardised QoL measures were administered.

Beran et al., 2009 [42]	CES-D	MOS-SF-36	Cytogenetic Testing Preferences Questionnaire			Psychological status did not vary as a function of cytogenetic test result. Nearly all participants, indicated that they wanted the prognostic information of the cytogenetic test, despite being informed that the result would not influence medical care.

Amaro et al., 2010 [33]	BDI	MOS-SF-36				In the QoL assessment, patients before the surgery showed a loss in the domain of role limitations owing to emotional problems. After 3 months, they described loss in vitality, social functioning and mental health. One year after surgery, recovery in the SF-36 scores.

Suchocka-Capuano et al., 2011 [34]	HADS	EORTC-QLQ-C30	QLQ-OPT-30	STAI-B		QoL levels remained relatively good and stable before and after treatment with the exception of social functioning. 56% of pts had moderate/severe anxiety that decreased after 1 month. Depressive symptoms remain stable

Cook et al., 2011 [43]		Audio-taped Interview				Patients who accepted cytogenetic test could not make a considered decision because of the emotionality of the situation. They were justifying their choice using normative ideas including altruism and the importance of being informed.

Wiley et al., 2013 [36]	CES-D	Concern about Recurrence Scale	VFQ-25			CES-D cutoff of 16 suggestive of clinical depression was 15.15%. This cohort reported high vision-specific QoL and low depressive symptoms.

Schuermeyer et al., 2016 [45]	HADS	Decision Regret Scale				The mean (SD) HADS anxiety score at baseline was higher than at 3 months or 12 months, and decreased with older age. The decision regret score was associated with baseline HADS depression score, and HADS depression score increased with baseline HADS anxiety score.

Hope-Stone et al., 2016 [46]	HADS	FACT-G				Female and younger patients showed higher levels of anxiety than other patients. Patients with monosomy 3 showed higher levels of depression. However, mean HADS scores remained below clinical relevance

## Abbreviations

UM: Uveal melanoma
CM: Choroidal melanoma
QoL: Quality of life
COMS: Collaborative Ocular Melanoma Study
DSM-IV-R: Diagnostic and Statistical Manual of Mental Disorders, IV Edition, Revised Version
DSM-III-R: Diagnostic and Statistical Manual of Mental Disorders, III Edition, Revised Version
HADS: The Hospital Anxiety and Depression Scale
BDI: Beck Depression Inventory
EORTC-QLQ: European Organization for Research and Treatment of Cancer Questionnaire
MOS: Medical Outcomes Study
MOS-SF-36: Medical Outcomes Study 36-item Short-Form Health Survey
ADVS: Activities of Daily Vision Scale
NEI-VFQ: National Eye Visual Functioning Questionnaire
QLQ-C30: European for Research and Treatment of Cancer Questionnaire
IES: Impact of Event Scale
MOOD: Measure of Outcome in Ocular Disease
SCL-90-R: Symptom Checklist 90 Revised
K22: Short Form of the Questionnaire for Social Support
GSI: Global Severity Index
PSDI: Positive Symptoms Distress Index
PST: Positive Symptoms Total
SEBI: Stereotactic external-beam irradiation
LINAC: 6-MV linear accelerator
GK: Leksell Gamma Knife therapy
VF-19: Visual Functioning Index modified to a 19-item version
EORTC QLQ-OPT30: European Organization for Research and Treatment of Cancer Questionnaire, Ophthalmic Oncology Quality of Life Questionnaire Module
STAI-B: State-Trait Anxiety Inventory
CNQ- SF: Cancer Needs Questionnaire-Short Form
NEI-VFQ: 25-item National Eye Institute Visual Function Questionnaire
CES-D: Center for Epidemiologic Studies Depression Scale
DRS: Decision Regret Scale
FACT-G: Functional Assessment of Cancer Therapy-General.
